# Supplementary material for: Structural and microstructural thalamocortical network disruption in sporadic behavioural variant frontotemporal dementia
Source: Neuroimage Clin. 2023 Jul 11;39:103471. doi: 10.1016/j.nicl.2023.103471 (PMC10371821; doi:10.1016/j.nicl.2023.103471)
Supplement: Supplementary data 1 [file mmc1.docx]

# Supplemental Information

Supplemental Table 1. Cortical thickness change between healthy control and bvFTD participants:

| Region | Left |  | Right |  |
| --- | --- | --- | --- | --- |
|  | Beta | p value | Beta | p value |
| bankssts | -0.125 | 0.0236 | -0.066 | 0.1875 |
| caudalanteriorcingulate | -0.204 | 0.0596 | -0.107 | 0.3341 |
| caudalmiddlefrontal | -0.132 | 0.0183 | -0.145 | 0.0075 |
| cuneus | -0.106 | 0.0179 | -0.023 | 0.5958 |
| entorhinal | -0.466 | 0.0062 | -0.395 | 0.0286 |
| fusiform | -0.104 | 0.0276 | -0.034 | 0.4850 |
| inferiorparietal | -0.068 | 0.0920 | -0.030 | 0.4753 |
| inferiortemporal | -0.157 | 0.0049 | -0.068 | 0.2021 |
| isthmuscingulate | -0.162 | 0.0186 | -0.231 | 0.0005 |
| lateraloccipital | 0.041 | 0.4164 | 0.065 | 0.1082 |
| lateralorbitofrontal | **-0.339** | **<0.0001** | **-0.239** | **0.0009** |
| lingual | -0.051 | 0.1882 | -0.018 | 0.6575 |
| medialorbitofrontal | -0.195 | 0.0122 | -0.268 | 0.0022 |
| middletemporal | -0.196 | 0.0022 | **-0.199** | **0.0011** |
| parahippocampal | -0.215 | 0.0993 | -0.177 | 0.0941 |
| paracentral | -0.020 | 0.7269 | -0.063 | 0.1804 |
| parsopercularis | -0.167 | 0.0054 | **-0.166** | **0.0014** |
| parsorbitalis | -0.274 | 0.0041 | **-0.238** | **0.0013** |
| parstriangularis | **-0.255** | **0.0003** | **-0.197** | **0.0011** |
| pericalcarine | -0.041 | 0.2227 | -0.033 | 0.3347 |
| postcentral | 0.004 | 0.9143 | -0.008 | 0.8403 |
| posteriorcingulate | **-0.178** | **0.0002** | -0.159 | 0.0161 |
| precentral | -0.122 | 0.0273 | -0.099 | 0.0159 |
| precuneus | -0.073 | 0.1416 | -0.040 | 0.3030 |
| rostralanteriorcingulate | **-0.334** | **0.0001** | -0.211 | 0.0356 |
| rostralmiddlefrontal | **-0.172** | **0.0061** | -0.181 | 0.0030 |
| superiorfrontal | **-0.184** | **0.0007** | **-0.219** | **0.0003** |
| superiorparietal | 0.006 | 0.8833 | 0.029 | 0.4927 |
| superiortemporal | -0.106 | 0.0941 | -0.137 | 0.0208 |
| supramarginal | -0.127 | 0.0047 | -0.054 | 0.2380 |
| frontalpole | -0.147 | 0.2087 | -0.227 | 0.0567 |
| temporalpole | -0.337 | 0.0149 | -0.208 | 0.0705 |
| transversetemporal | -0.051 | 0.5420 | 0.029 | 0.7396 |
| insula | **-0.278** | **<0.0001** | -0.135 | 0.0129 |

Highlighted regions are significant at a Bonferroni-like corrected threshold of 0.0015 (0.05/34).

Supplemental Table 2. Cortical thickness correlations with clinical and behavioural measures.

| Region | FTLD |  |  |  | FBI all |  |  |  |
| --- | --- | --- | --- | --- | --- | --- | --- | --- |
|  | Right |  | Left |  | Right |  | Left |  |
|  | Beta | p | Beta | p | Beta | p | Beta | p |
| bankssts | -0.010 | 0.280 | -0.004 | 0.394 | -0.004 | 0.382 | -0.004 | 0.394 |
| caudalanteriorcingulate | -0.023 | 0.404 | -0.013 | 0.259 | -0.007 | 0.591 | -0.013 | 0.259 |
| caudalmiddlefrontal | -0.022 | 0.053 | -0.002 | 0.791 | -0.012 | 0.036 | -0.002 | 0.791 |
| cuneus | 0.001 | 0.957 | 0.001 | 0.747 | 0.005 | 0.253 | 0.001 | 0.747 |
| entorhinal | -0.019 | 0.627 | -0.028 | 0.099 | 0.000 | 0.989 | -0.028 | 0.099 |
| fusiform | -0.009 | 0.415 | 0.001 | 0.842 | -0.003 | 0.597 | 0.001 | 0.842 |
| inferiorparietal | 0.005 | 0.559 | -0.005 | 0.271 | 0.004 | 0.306 | -0.005 | 0.271 |
| inferiortemporal | -0.002 | 0.855 | -0.002 | 0.689 | 0.002 | 0.700 | -0.002 | 0.689 |
| isthmuscingulate | -0.016 | 0.136 | -0.003 | 0.624 | -0.003 | 0.600 | -0.003 | 0.624 |
| lateraloccipital | 0.012 | 0.141 | 0.005 | 0.375 | 0.006 | 0.133 | 0.005 | 0.375 |
| lateralorbitofrontal | -0.037 | 0.009 | -0.007 | 0.399 | -0.004 | 0.547 | -0.007 | 0.399 |
| lingual | 0.000 | 0.985 | 0.002 | 0.716 | 0.000 | 0.926 | 0.002 | 0.716 |
| medialorbitofrontal | -0.032 | 0.066 | 0.001 | 0.951 | 0.002 | 0.860 | 0.001 | 0.951 |
| middletemporal | 0.005 | 0.724 | -0.002 | 0.805 | 0.005 | 0.500 | -0.002 | 0.805 |
| parahippocampal | -0.024 | 0.313 | -0.020 | 0.114 | -0.015 | 0.179 | -0.020 | 0.114 |
| paracentral | -0.016 | 0.074 | -0.006 | 0.343 | -0.006 | 0.205 | -0.006 | 0.343 |
| parsopercularis | -0.021 | 0.045 | 0.002 | 0.805 | -0.002 | 0.725 | 0.002 | 0.805 |
| parsorbitalis | -0.037 | 0.004 | -0.007 | 0.481 | -0.006 | 0.334 | -0.007 | 0.481 |
| parstriangularis | -0.021 | 0.045 | -0.005 | 0.472 | -0.005 | 0.318 | -0.005 | 0.472 |
| pericalcarine | -0.007 | 0.253 | 0.000 | 0.935 | -0.001 | 0.841 | 0.000 | 0.935 |
| postcentral | 0.000 | 0.975 | 0.000 | 0.919 | 0.002 | 0.685 | 0.000 | 0.919 |
| posteriorcingulate | -0.031 | 0.020 | -0.003 | 0.466 | -0.009 | 0.163 | -0.003 | 0.466 |
| precentral | -0.014 | 0.078 | -0.011 | 0.061 | -0.008 | 0.030 | -0.011 | 0.061 |
| precuneus | -0.007 | 0.451 | -0.004 | 0.454 | -0.002 | 0.717 | -0.004 | 0.454 |
| rostralanteriorcingulate | -0.033 | 0.168 | -0.007 | 0.278 | -0.001 | 0.953 | -0.007 | 0.278 |
| rostralmiddlefrontal | -0.010 | 0.391 | 0.000 | 0.964 | 0.002 | 0.762 | 0.000 | 0.964 |
| superiorfrontal | -0.029 | 0.024 | -0.006 | 0.243 | -0.009 | 0.112 | -0.006 | 0.243 |
| superiorparietal | -0.001 | 0.919 | -0.001 | 0.782 | 0.001 | 0.907 | -0.001 | 0.782 |
| superiortemporal | -0.005 | 0.712 | -0.005 | 0.542 | -0.001 | 0.851 | -0.005 | 0.542 |
| supramarginal | -0.006 | 0.523 | -0.004 | 0.329 | -0.002 | 0.659 | -0.004 | 0.329 |
| frontalpole | -0.003 | 0.890 | 0.008 | 0.508 | 0.011 | 0.250 | 0.008 | 0.508 |
| temporalpole | -0.004 | 0.868 | 0.009 | 0.563 | -0.001 | 0.971 | 0.009 | 0.563 |
| transversetemporal | -0.007 | 0.596 | -0.006 | 0.429 | -0.008 | 0.211 | -0.006 | 0.429 |
| insula | -0.001 | 0.962 | -0.004 | 0.603 | 0.002 | 0.769 | -0.004 | 0.603 |

Supplemental Table 2. Cortical thickness correlations with clinical and behavioural measures (cont).

|  | FBI 1-10 |  |  |  | FBI 12-20 |  |  |  |
| --- | --- | --- | --- | --- | --- | --- | --- | --- |
|  | Right |  | Left |  | Right |  | Left |  |
| Region | Beta | p | Beta | p | Beta | p | Beta | p |
| bankssts | -0.004 | 0.535 | -0.003 | 0.674 | -0.004 | 0.633 | -0.007 | 0.436 |
| caudalanteriorcingulate | 0.004 | 0.854 | -0.021 | 0.196 | -0.043 | 0.107 | -0.009 | 0.720 |
| caudalmiddlefrontal | -0.009 | 0.264 | -0.003 | 0.732 | -0.023 | 0.031 | 0.000 | 0.977 |
| cuneus | 0.008 | 0.229 | 0.001 | 0.836 | 0.003 | 0.707 | 0.001 | 0.945 |
| entorhinal | -0.005 | 0.857 | -0.044 | 0.058 | -0.005 | 0.902 | -0.030 | 0.389 |
| fusiform | 0.002 | 0.809 | 0.000 | 0.985 | -0.016 | 0.140 | 0.003 | 0.804 |
| inferiorparietal | 0.006 | 0.296 | -0.008 | 0.186 | 0.005 | 0.556 | -0.002 | 0.828 |
| inferiortemporal | 0.002 | 0.732 | -0.001 | 0.883 | 0.000 | 0.966 | -0.009 | 0.419 |
| isthmuscingulate | -0.002 | 0.734 | -0.004 | 0.633 | -0.006 | 0.519 | 0.000 | 0.986 |
| lateraloccipital | 0.011 | 0.050 | 0.008 | 0.310 | 0.000 | 0.958 | 0.003 | 0.771 |
| lateralorbitofrontal | -0.003 | 0.768 | -0.010 | 0.386 | -0.011 | 0.399 | -0.007 | 0.673 |
| lingual | 0.001 | 0.844 | 0.001 | 0.911 | -0.005 | 0.641 | 0.005 | 0.560 |
| medialorbitofrontal | 0.006 | 0.629 | -0.003 | 0.832 | -0.013 | 0.462 | 0.001 | 0.962 |
| middletemporal | 0.006 | 0.492 | -0.003 | 0.737 | 0.003 | 0.815 | -0.003 | 0.790 |
| parahippocampal | -0.021 | 0.184 | -0.039 | 0.024 | -0.026 | 0.249 | -0.010 | 0.694 |
| paracentral | -0.007 | 0.285 | -0.014 | 0.085 | -0.007 | 0.417 | 0.010 | 0.430 |
| parsopercularis | -0.002 | 0.825 | 0.003 | 0.736 | -0.004 | 0.730 | 0.001 | 0.914 |
| parsorbitalis | -0.008 | 0.373 | -0.013 | 0.352 | -0.006 | 0.643 | 0.002 | 0.936 |
| parstriangularis | -0.007 | 0.360 | -0.009 | 0.342 | -0.005 | 0.611 | 0.001 | 0.924 |
| pericalcarine | -0.002 | 0.644 | -0.001 | 0.875 | 0.001 | 0.826 | 0.001 | 0.757 |
| postcentral | 0.002 | 0.733 | 0.003 | 0.664 | 0.001 | 0.881 | -0.005 | 0.603 |
| posteriorcingulate | -0.010 | 0.297 | -0.008 | 0.102 | -0.020 | 0.135 | 0.006 | 0.388 |
| precentral | -0.010 | 0.049 | -0.019 | 0.018 | -0.008 | 0.327 | 0.000 | 0.978 |
| precuneus | -0.002 | 0.749 | -0.007 | 0.295 | -0.001 | 0.878 | 0.002 | 0.837 |
| rostralanteriorcingulate | 0.005 | 0.704 | -0.013 | 0.171 | -0.026 | 0.191 | -0.002 | 0.881 |
| rostralmiddlefrontal | 0.006 | 0.370 | 0.000 | 0.981 | -0.009 | 0.374 | 0.001 | 0.907 |
| superiorfrontal | -0.005 | 0.483 | -0.005 | 0.463 | -0.020 | 0.053 | -0.009 | 0.340 |
| superiorparietal | -0.001 | 0.816 | 0.001 | 0.832 | 0.006 | 0.489 | -0.006 | 0.501 |
| superiortemporal | 0.000 | 0.955 | -0.006 | 0.527 | -0.003 | 0.799 | -0.004 | 0.762 |
| supramarginal | -0.004 | 0.547 | -0.007 | 0.263 | 0.000 | 0.976 | 0.000 | 0.960 |
| frontalpole | 0.009 | 0.522 | 0.003 | 0.862 | 0.019 | 0.325 | 0.021 | 0.377 |
| temporalpole | 0.002 | 0.908 | 0.002 | 0.940 | -0.011 | 0.684 | 0.018 | 0.547 |
| transversetemporal | -0.009 | 0.352 | -0.006 | 0.585 | -0.014 | 0.297 | -0.013 | 0.419 |
| insula | 0.004 | 0.598 | -0.006 | 0.568 | -0.008 | 0.483 | -0.007 | 0.638 |

Supplemental Table 3. DTI correlations with clinical and behavioural measures.

|  | FTLD |  |  | FBI total |  |  | FBI 1-10 |  |  | FBI 12-20 |  |  |
| --- | --- | --- | --- | --- | --- | --- | --- | --- | --- | --- | --- | --- |
|  | Beta | SE | p | Beta | SE | p | Beta | SE | p | Beta | SE | p |
| FA |  |  |  |  |  |  |  |  |  |  |  |  |
| Left ATR | -0.005 | 0.004 | 0.199 | -0.002 | 0.002 | 0.385 | -0.002 | 0.003 | 0.487 | -0.002 | 0.003 | 0.487 |
| Right ATR | **-0.010** | **0.004** | **0.014** | -0.003 | 0.002 | 0.214 | -0.005 | 0.003 | 0.099 | -0.005 | 0.003 | 0.099 |
| Left PTR | -0.002 | 0.003 | 0.446 | -0.002 | 0.001 | 0.246 | **-0.004** | **0.002** | **0.049** | **-0.004** | **0.002** | **0.049** |
| Right PTR | -0.006 | 0.003 | 0.087 | **-0.004** | **0.001** | **0.023** | **-0.005** | **0.002** | **0.026** | **-0.005** | **0.002** | **0.026** |
| MD (× 10^-4^) |  |  |  |  |  |  |  |  |  |  |  |  |
| Left ATR | 0.062 | 0.025 | 0.258 | 0.024 | 0.013 | 0.115 | 0.031 | 0.019 | 0.192 | 0.031 | 0.019 | 0.192 |
| Right ATR | 0.031 | 0.027 | 0.258 | 0.014 | 0.013 | 0.308 | 0.025 | 0.018 | 0.192 | 0.025 | 0.018 | 0.192 |
| Left PTR | 0.034 | 0.019 | 0.088 | 0.017 | 0.010 | 0.115 | 0.025 | 0.014 | 0.079 | 0.025 | 0.014 | 0.079 |
| Right PTR | 0.039 | 0.022 | 0.094 | **0.024** | **0.011** | **0.044** | **0.034** | **0.015** | **0.036** | **0.034** | **0.015** | **0.036** |

## Validation analyses

SPHARM-PDM (Styner et al., 2006) was used as an alternative shape analysis methodology. Briefly, the segmentations were minimally smoothed with a 1 mm Gaussian kernel and internal holes filled to ensure a spherical topology. Smoothed images were described by spherical harmonics to determine correspondence and then sampled into meshes with 1002 vertices. Surfaces were aligned using a rigid-body Procrustes alignment to a study-specific mean template for each structure. Finally, we calculated the displacement of each vertex for each subject from the mean shape along the vertex normal. These vertex displacement measures were used as the dependent variable in subsequent analyses.

Initially, we utilised SPHARM-PDM vertex analysis as described in the original publication. Here, linear models were analysed at each vertex (vertex displacement ~ ICV + Age + Group) to yield 1002 coefficients and p-values. P-values were subsequently corrected for multiple comparisons using FDR correction. Coefficients are presented in the top left of Supplemental Figure S1. No vertices were significant after FDR correction (not shown).

Subsequently, vertex displacement measures were used in PLS-DA analysis, with control of ICV and age as detailed in the manuscript. Results of coefficients are presented in the top right of Supplemental Figure S1.

Next, we used Deformetrica shape analyses. Here shapes were processed as detailed in the manuscript and two types of analyses were performed. First, like SPHARM-PDM vertex analyses, we created linear models for each momenta direction vector controlling for ICV and age and comparing groups. The coefficient for the group comparison was then projected along the existing momenta directions (“shooting”, in Deformetrica terms) to deform the average model. For visualisation, we observed the displacement along the normal vector between the average and the projected shape, shown in the top right of Supplemental Figure S1. Lastly, we used the momenta as above for PLS-DA classification for the thalami, projected the loadings as described in the manuscript, and visualised the results in the bottom right of Supplemental Figure S1.

The results of various shape analysis and imaging methods are similar in visual comparison.

Supplemental Figure 1.

Group differences using various shape and statistical analyses methods.


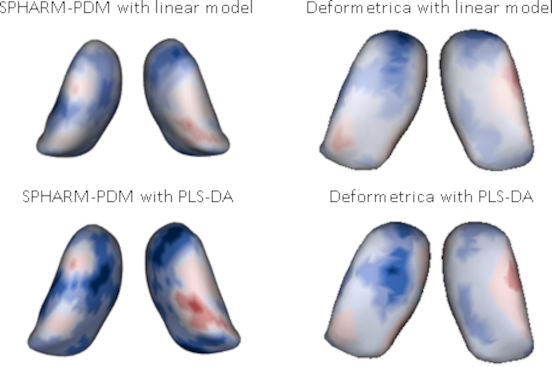


Views of bilateral thalami from superior/top view. Colours indicate displacement relative to an average shape; cooler colours mean inward displacement of the surface, and warmer colours are outward displacement of the surface. Absolute scales are centred around zero with arbitrary magnitude.

## Alternative cortical metrics

We initially used cortical thickness in all analyses, but subsequently assessed models using cortical volume, area, and mean curvature. Apart from different cortical metrics the analyses methodology was identical as the current analyses. Results of volume, area, and curvature analyses are presented below in Figures R1, R2, and R3 respectively.

Using cortical volume, sparsity tuning selected 50 cortical, 6 tracts, and 100 thalamic deformation points. Model sensitivity was 92%, specificity 68%, p-value = 0.0495. Notably, the most anterior cortical regions were not selected, but higher weighting was applied to the primary motor area.

The area metric selected only 10 cortical areas, 7 tracts, and 120 thalamic deformation points. Model sensitivity was 92%, specificity 77%, p-value = 0.010. Only a few cortical areas were selected, involving reduced area in the right primary motor and sensory cortex, whereas mildly increased area in the left motor and sensory areas.

Curvature sparsity selected only 12 cortical regions, 2 tracts, and 250 thalamic deformation points. Sensitivity 92%, specificity 68%, p-value = 0.030. Few cortical regions were selected. Notably few cortical regions were selected.

Results for diffusion and thalamic morphology are generally consistent. The MD of the anterior thalamic radiations and anterior thalamic changes were consistently the most salient features differentiating groups regardless of cortical metrics used.

Figure R1. Sparse PLS-DA group comparison using cortical volume


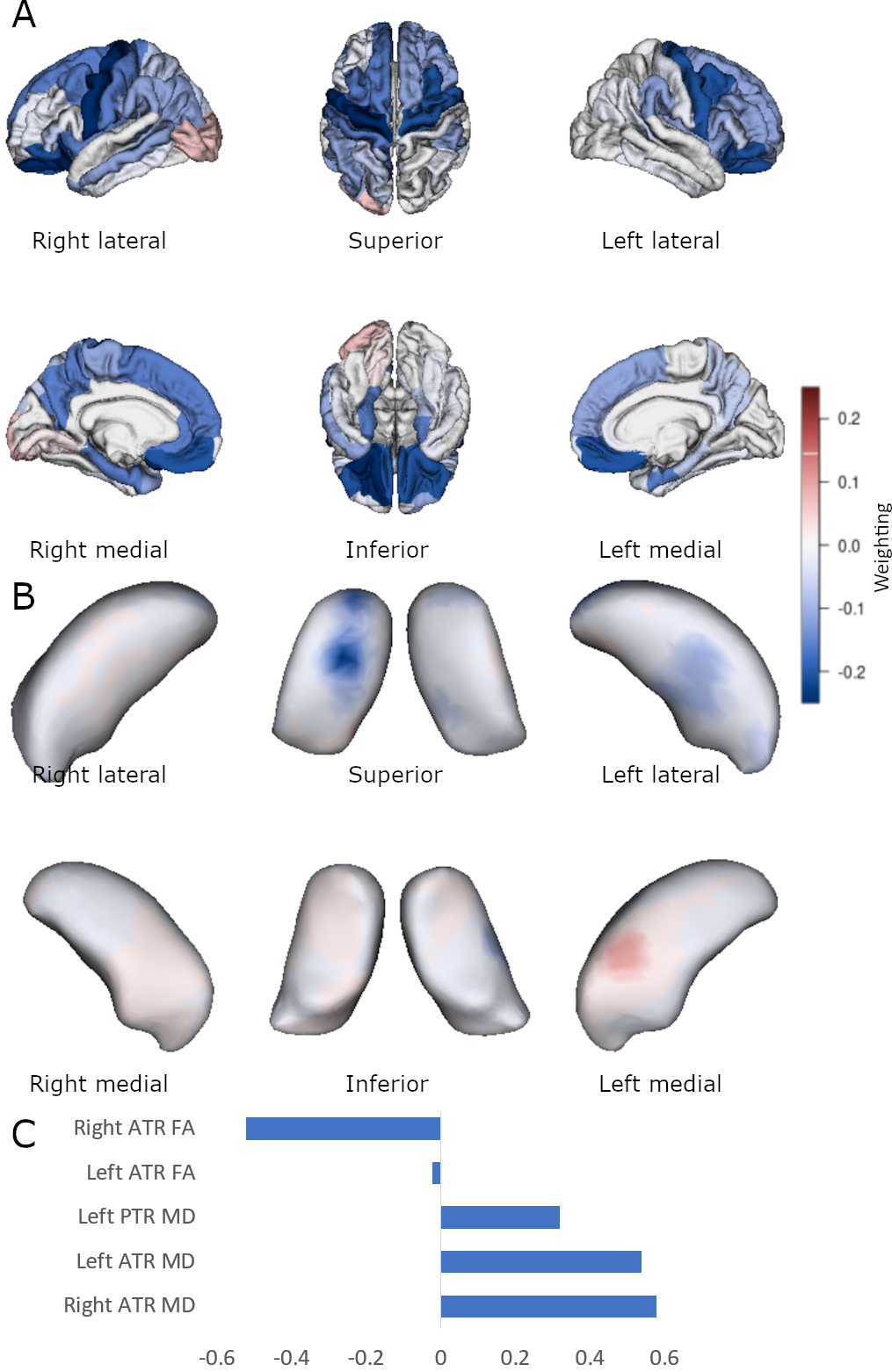


Legend: Multiblock group comparison. Panel A shows cortical volume loading values for the sparse model. Panel B shows displacement from an average thalamic shape after deformation by sparsely selected momenta. Panel C shows sparsely selected DTI tracts. ATR, anterior thalamic radiation; PTR, posterior thalamic radiation; FA, fractional anisotropy; MD, mean diffusivity. Scales show relative weighting in the discrimination selection model and are comparable in colour between Panels A and B.

Figure R2. Sparse PLS-DA group comparison using cortical area


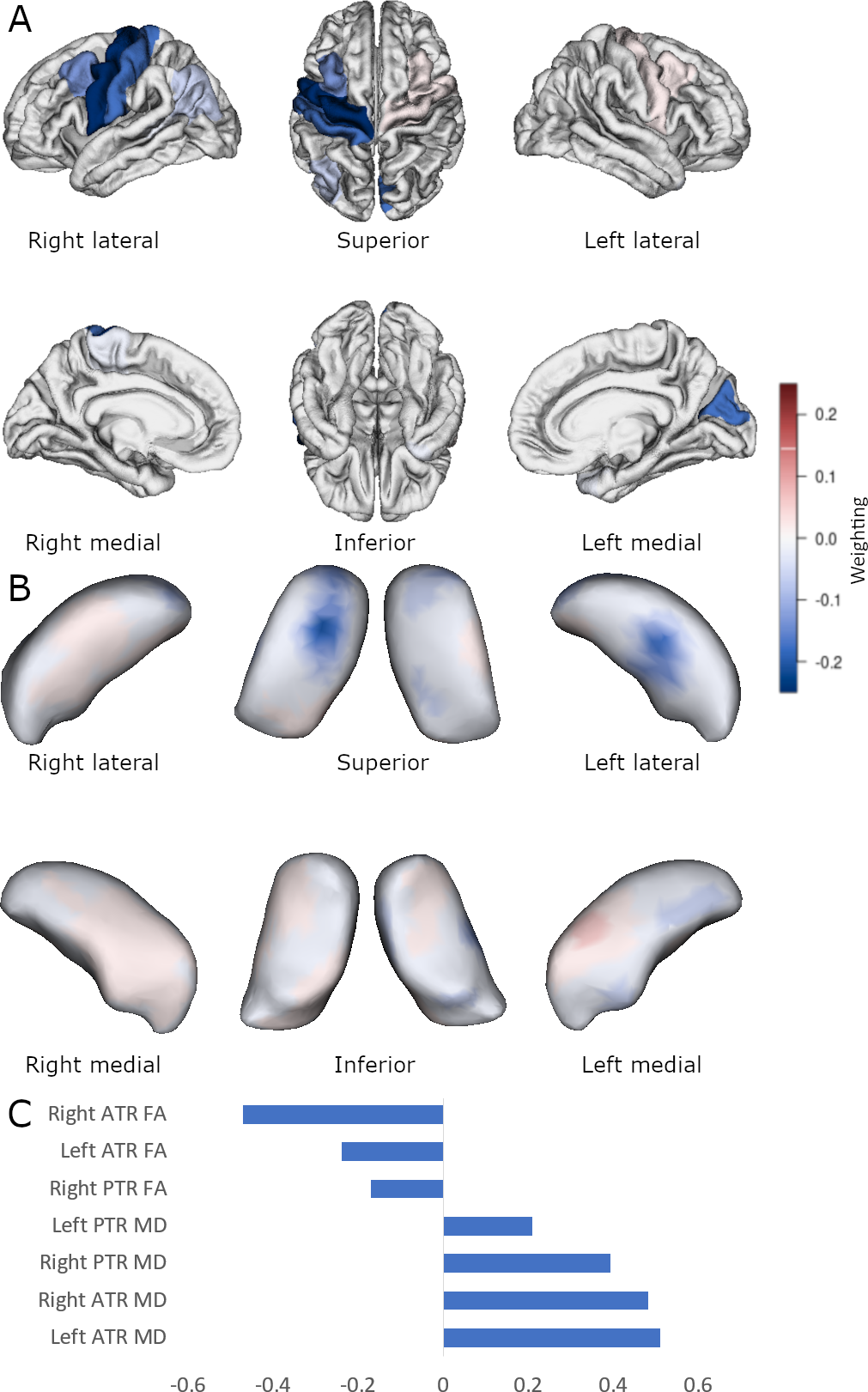


Legend: Multiblock group comparison. Panel A shows cortical area values for the sparse model. Panel B shows displacement from an average thalamic shape after deformation by sparsely selected momenta. Panel C shows sparsely selected DTI tracts. ATR, anterior thalamic radiation; PTR, posterior thalamic radiation; FA, fractional anisotropy; MD, mean diffusivity. Scales show relative weighting in the discrimination selection model and are comparable in colour between Panels A and B.

Figure R1. Sparse PLS-DA group comparison using cortical curvature


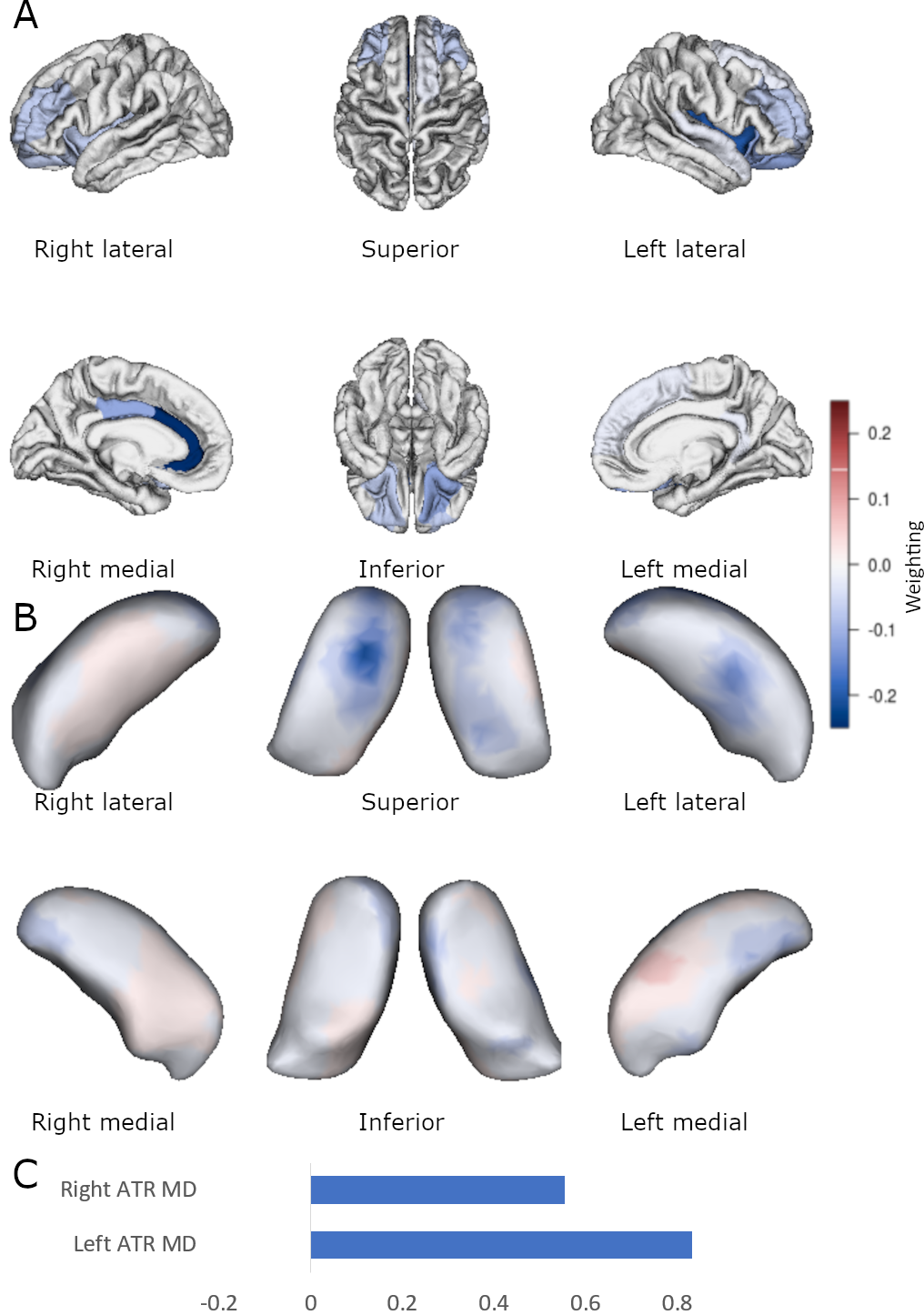


Legend: Multiblock group comparison. Panel A shows cortical curvature loading values for the sparse model. Panel B shows displacement from an average thalamic shape after deformation by sparsely selected momenta. Panel C shows sparsely selected DTI tracts. ATR, anterior thalamic radiation; PTR, posterior thalamic radiation; FA, fractional anisotropy; MD, mean diffusivity. Scales show relative weighting in the discrimination selection model and are comparable in colour between Panels A and B.
